# Resource planning principles for the radiotherapy process using simulations applied to a longer vacation period use case

**DOI:** 10.1016/j.tipsro.2021.10.001

**Published:** 2021-10-16

**Authors:** Jesper Lindberg, Mrugaja Gurjar, Paul Holmström, Stefan Hallberg, Thomas Björk-Eriksson, Caroline E Olsson

**Affiliations:** a Medical Radiation Sciences, Institute of Clinical Sciences, Sahlgrenska Academy, University of Gothenburg, Sweden; b Department of Medical Physics and Biomedical Engineering, Sahlgrenska University Hospital, Gothenburg, Sweden; c Regional Cancer Centre West, Western Sweden Healthcare Region, Gothenburg, Sweden; d Department of Oncology, Institute of Clinical Sciences, Sahlgrenska Academy, University of Gothenburg, Sweden

**Keywords:** Radiotherapy, Resource planning, Vacation, Simulation

## Abstract

**Purpose:**

Radiotherapy (RT) resources need to be used wisely to balance workload and patient throughput. There are no known strategies on how to plan resource use around longer vacation periods to avoid patient waiting times. We created a simulation model over the RT workflow to evaluate different scenarios for this purpose.

**Materials and Methods:**

The simulation model mimics a large modern RT department in Sweden. It was based on real data on patient referral patterns and resource use extracted from clinical systems (3666 treatment courses). Workshops with managers and staff were held to investigate nine different scenarios for the summer vacation period including one scenario to validate the model. Different capacity reductions, vacation period lengths and timing of the vacation periods between the preparatory part of the RT workflow and the treatment part were evaluated.

**Results:**

For an eight-week period, resource utilization was predicted to be high both before and after the vacation period regardless of timing. However, more patients would be waiting with completed preparations with simultaneous vacation periods than when the preparatory part started one-two weeks prior to the treatment part. With shorter vacation periods, treatment would require overtime during the vacation period with higher levels of patients waiting compared to an eight-week scenario.

**Conclusions:**

Our proposed strategy aided managers to identify a preferred scenario for the summer vacation period with the preparatory part starting one-two weeks prior to the treatment part for an eight-week vacation period. This can help other RT departments to plan for similar situations.

## Highlights

• An RT process simulation model aided managers with resource planning decisions.
• Several scenarios could easily be evaluated without affecting the clinical work.
• Starting vacation periods for preparations prior to treatment was most beneficial.

## Introduction

The global cancer incidence is increasing, with the consequence of an increased demand for radiotherapy (RT) [1]. In addition, the current use of RT is lower than the predicted optimal level, thus there are patients currently not receiving RT but who would benefit from this treatment [2]. Already, long patient waiting times is a reality at many RT departments and a common reason for this is lack of resources, both equipment and staff [3]. Resource planning in RT is important to establish or maintain short waiting times as long waiting times to RT can impact clinical outcomes and also can cause high levels of anxiety for the patient, their relatives, and caregivers [4]. Patients waiting for treatment has also been identified as a factor to cause stress for RT staff which, in turn, can compromise quality and safety of the treatments [5]. Waiting times can occur both between referral and start of RT preparations as well as between completed preparations and start of treatment. From the staff perspective, resource planning is also important to assure that sufficient time can be spent on quality programs as well as to enable continuous professional development to advance practice. The available resources must, therefore, be used in the best possible way without jeopardizing clinical outcomes, without exhausting RT staff, and at the same time handle the expected increase in referrals. This means that the RT community must be open for new working methods to meet future challenges, but the already high workload in RT limits the possibilities to evaluate and implement such strategies.

To enable evaluation of different resource planning scenarios, simulation models can be a valuable tool [6]. A variety of strategies for handling a specific scenario can be assessed, without influencing daily work, which can be particularly useful when wanting to evaluate new working methods in a busy healthcare environment before implementing changes in reality. Simulation results from the testing of different strategies contributes to an increased understanding of underlying effects and can inform managers’ decisions regarding resource planning in both the short and the long perspective. In RT, different operations research methods including simulation models have previously been applied to optimize patient scheduling and capacity planning to identify best-case solutions for specific situations [7]. However, none have yet explored the ability of simulation models to address effects on the complete RT workflow by different resource planning policies in a more general sense.

The aim of this work was to use continuous simulation methodology to create a model of the complete RT process and to investigate if this could aid RT managers in taking informed decisions about how to plan for resource use. Different scenarios for the summer vacation period, simulated based on real data from at a large modern RT department in Sweden, were evaluated as a use case together with RT managers and staff.

## Materials and methods

### RT department and workflow

The RT department that the simulation model mimics is located at one of Sweden’s University hospitals. The department is one of the three largest in Sweden with a catchment area of approximately one fifth of the Swedish population. It consists of two sites which currently have 11 linear accelerators (linacs), two computed tomography (CT) scanners and one magnetic resonance imaging (MRI). This study uses information from the main site which had 9 operating linacs at the time when referral data for the simulation model was collected and 10 operating linacs at the time of the actual simulations. Around 150 RT staff (primarily nurses specializing in oncology) work at the department and treat all types of cancer, both with curative and palliative intent. Typical opening hours for the department is 8 h and 45 min per day, Monday-Friday. The RT workflow consists of positioning aid (mould), imaging (CT, MRI and positron-emission tomography [PET]), contouring, treatment planning, patient quality assurance (QA) and treatment. At treatment, patient receives the radiation, typically once per day during several weeks but number of treatment fractions varies between treatment intent and diagnosis. Daily staffing of the investigated department (working within the RT workflow) typically engage about 25 nurses at pre-treatment (1–2 at mould, 3 at each CT; 2 at MR and 15 at treatment planning) and about 4 nurses per treatment room; there are about 7 medical physicists and 9 oncologists including residents working across the RT workflow. The patients are scheduled for both pre-treatment and treatment activities as the referral to RT reaches the department. In the simulation model, treatment begins as soon the patient completed the preparations if there is available linac capacity. RT organization and work situation in Sweden have recently been described [3], [8]. In short, nurses (RT nurses are specially educated and comparable to radiotherapy technologists) are typically stationed at a specific workflow step, either at a pre-treatment task or at treatment, while medical physicists and oncologists work across the whole workflow. This also applies to the investigated department.

### Simulation model development and data extraction

Model development and simulations were conducted in Stella Architect (versions 1.7.1–2.1, isee systems, Lebanon, NH, U.S.A.), in close collaboration between experienced modelers (PH, SH, and MG) and experts in RT (JL, TBE, and CO). Regular meetings with managers and staff responsible for patient scheduling was also held to validate and focus development to the department’s clinical needs. Model validation included evaluations of model structure (ensuring that patient flows in the model were consistent and expected), test of extreme values (ensuring that very low/high capacity or low/high patient inflow resulted in expected model behavior) and a third-party face validation by the managers and staff at the investigated RT department where historical patterns were visually validated. Detailed descriptions of the simulation model and user interface are given in supplementary material.

Real patient data was retrieved from the department’s oncology information system, ARIA (Varian Medical Systems, Inc., Palo Alto, CA, U.S.A.), for the largest site and the period January 1st, 2015 to April 30th, 2016 (70 weeks). The retrieved information included referral date, diagnosis, intent, use of preparatory resources (set to 1.5 weeks, the typical time span from mould to QA at the investigated department during the time period of the study) and treatment resources (number of treatment fractions, for each patient). Data was manually pre-processed as described in detail in a previous study [9]. In short, this included the removal of duplicate and non-logical bookings as well as organizing the information in groups by diagnosis and treatment intent to arrive at a suitable input data format for the simulation model according to the Pareto principle. The Pareto principle was used to acknowledge all major patient groups without inflating model complexity and can be expected to result in more realistic simulations of RT workflow effects than, for instance, using a group average over all patients as input [9].

### Simulated use case and scenarios

In Sweden, RT employees have a legislated right of four successive weeks of vacation during June to August [10]. This annually recurring period of capacity reduction needs to be well planned to prohibit buildup of long patient waiting times whilst assuring that all staff have their vacation. Timing of staff leave needs to be balanced with patients’ incoming referrals and ongoing treatments. The data set used as basis to simulate different policies around this longer vacation period were sorted in weekly batches and scaled to meet the patient volumes of 2020 for the whole department. To identify a favorable scenario for how to use available resources during the summer vacation period, different levels of resource reduction and timing between vacation periods of the preparatory part and the treatment part were tested in the simulation model. Baseline capacities (simulated capacity outside vacation period) for the two parts were set to the lowest level not building persistent queues, for an easier comparison of scenarios. The simulated time period was 70 weeks, equally long as the period for the collected data. The patients’ first treatment fraction was set to require twice the amount of linac capacity compared to subsequent fractions, since more time is needed for the staff to inform the patient about the treatment and to verify technical details at this first occasion. RT workflow details on treatment technique and specific patient positioning requirements was not included in the simulation model, neither was linac maintenance/downtime. Specific requirements or other policies influencing the RT workflow was assumed to have equal impact during the vacation period and when running at normal capacity. Model output data display number of patients.

## Results

### Input data overview

The collected data from 2015 to 2016 (70 weeks) included a total number of 3209 patients referred for RT (scheduled for 3666 treatment courses). Of the referred patients, 2094 (65%) were planned for treatment with curative intent and 1115 (35%) with palliative intent, with a total of 128 different RT workflows. By aggregating the data by the 80/20 Pareto principle number of workflows was reduced to 21. A scaling factor of 1.7 were used to meet the number of courses scheduled for treatment in 2020 (4610 patients in 53 weeks).

### Summer vacation period

The baseline capacity used in the simulation model for the preparation part was set to 100 patients per week and for the treatment part to 330 fractions per day. Simulations were initiated with settings similar to the vacation period for the previous year (2020) to face-validate the model (Scenario #1, Table 1). The output from these simulations were confirmed with the participants to correspond to their experienced situation at that time and aroused their curiosity for further explorations. Altogether, four managers and two staff responsible for the scheduling task participated in two workshops held three weeks apart in April 2021. The additional scenarios were adapted to what the participants wanted to explore (Scenario #2–9, Table 1). In total, nine different scenarios were evaluated, with three different period lengths, eight, six, and four weeks, results for each scenario are described below.

**Table 1:** Simulated capacity scenarios during the summer vacation period. Capacity reduction for the first scenario corresponds to the same range of capacity reduction used for the department in 2020.

Scenario	Preparation part in relation to treatment part	Period length (weeks)	Capacity reduction	
			Preparation	Treatment
#1	Simultaneous	8	30%	30%
#2	Simultaneous	8	50%	30%
#3	Four weeks earlier	8	30%	30%
#4	Two weeks earlier	8	30%	30%
#5	One week earlier	8	30%	30%
#6	Simultaneous	6	50%	50%
#7	One week earlier	6	50%	50%
#8	One week earlier	4	70%	70%
#9	Four weeks earlier	4	70%	70%

#### Eight-week vacation period

**Fig. 1:**
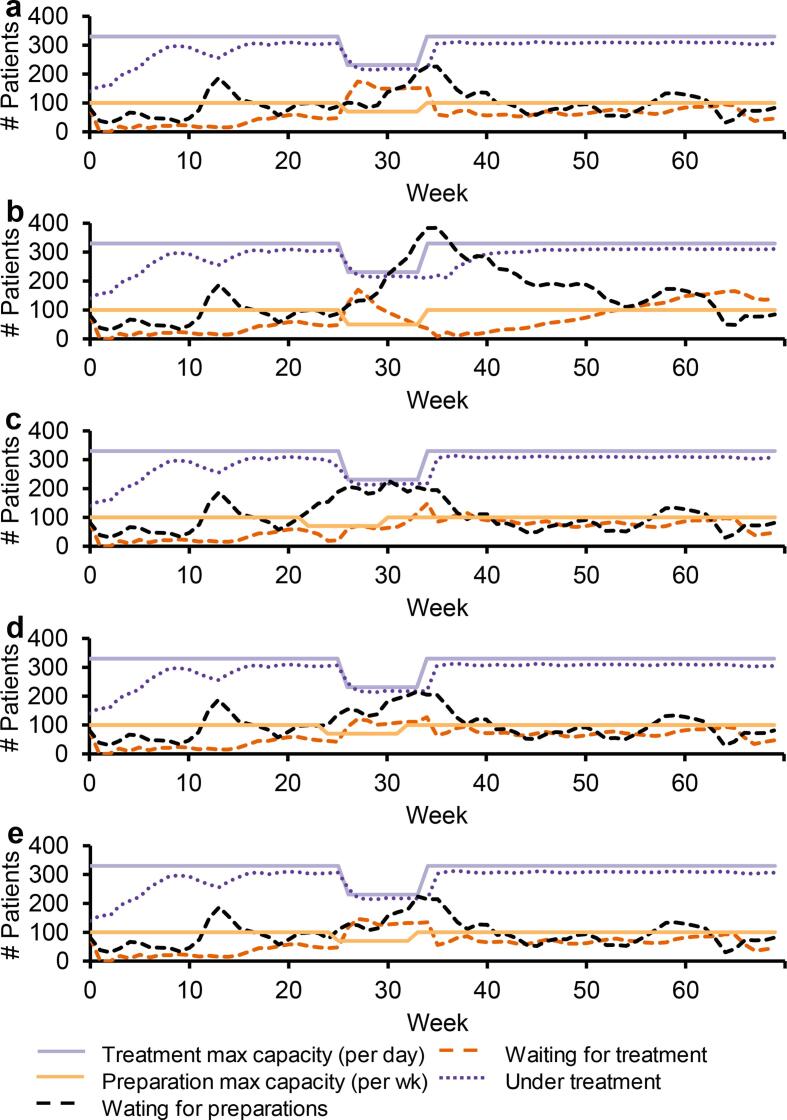
Capacities and simulation results for radiotherapy preparation and treatment steps during an eight-week summer vacation period. Simultaneous capacity reduction of both parts with 30% (a) and with 50% and 30% capacity reductions for preparation and treatment, respectively (b). Capacity reduction of 30% for both parts with the preparation vacation period starting four (c), two (d), one (e) week prior the treatment vacation period.

Simulations of a simultaneous capacity reduction of 30% for both workflow parts resulted in a buildup of patients waiting to start treatment having completed preparations. After the vacation period, a high utilization of linacs was possible (Scenario 1; Fig. 1a). With additional capacity reduction of the preparation part to 50% whilst keeping the capacity reduction for the treatment part to 30%, the number of patients with completed preparations waiting to start treatment was reduced, however, there were too few patients available to start treatment after the vacation period, making the treatment part underutilized for several weeks (Scenario 2; Fig. 1b).

Moving the vacation period for the preparation part to four weeks prior to the vacation period for the treatment part, but keeping the capacity reduction to 30% for both, resulted in fewer patients waiting to start treatment with completed preparations compared to the previous scenarios. This scenario had enough patients to utilize treatment capacity after the vacation period, however, with an underutilization before the treatment vacation period started (Scenario 3; Fig. 1c). With vacation periods of the preparation part instead starting one and two weeks prior to the vacation period of the treatment part, fewer patients were waiting compared to the first two scenarios. These scenarios had enough patients before and after the vacation period to utilize treatment capacity, but with a need for working overtime the first week of the treatment vacation period (Scenario 4–5; Fig. 1d-e).

#### Six-week vacation period

**Fig. 2:**
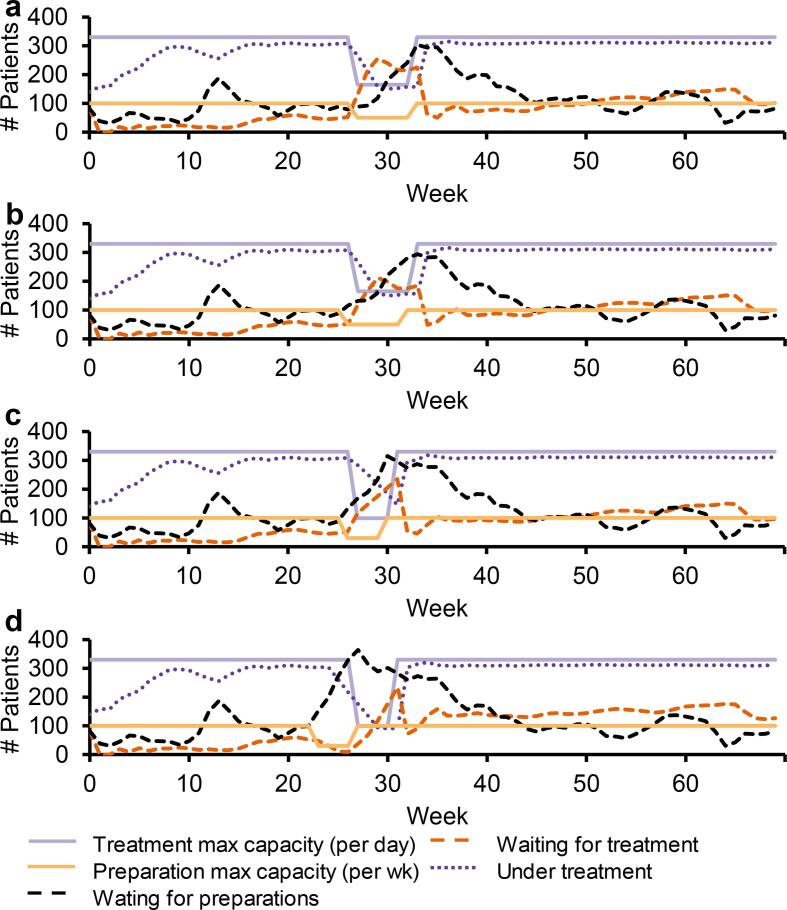
Capacities and simulation results for radiotherapy preparation and treatment steps during a six-week or four-week summer vacation period. For a six-week long vacation period with a simultaneous capacity reduction of both parts with 50% (a) and still with 50% reduction but with the preparatory vacation period moved one week prior to the treatment part vacation period (b). For a four-week long vacation period with 70% capacity reduction for both parts with the preparatory vacation period moved one (c) and four (d) week prior to the treatment part vacation period.

With a simultaneous capacity reduction of 50%, simulations resulted in more patients waiting with completed preparations compared to previous scenarios and a higher demand for overtime at treatment the first week of the vacation period (Scenario 6; Fig. 2a). When moving the vacation period for the preparation part to one week prior to the vacation period for the treatment part whilst keeping the capacity reduction to 50% for both parts, fewer patients were waiting with completed preparations compared to the simultaneous reduction (Scenario 7; Fig. 2b).

#### Four-week vacation period

A simultaneous capacity reduction of 70% and having the vacation period for the preparatory part starting one week earlier than the vacation period for the treatment part resulted in a need for overtime at treatment for the whole vacation period (Scenario 8; Fig. 2c). Moving the vacation period for the preparation part four weeks earlier, whilst keeping the capacity reduction to 70% for both parts, ending when treatment vacation period began, resulted in lower need of overtime for the treatment part compared to the previous scenario but with an underutilization at treatment before the treatment vacation period (Scenario 9; Fig. 2d).

## Discussion

In this work, we investigated different resource planning scenarios using a novel simulation model over the whole RT process. Our aim was to develop a simulation model and to evaluate if this model could aid managers in taking informed decisions about how to avoid overtime for staff, build-up of patients waiting to initiate treatment and maximize linac use when planning for reduced capacity over a longer vacation period. We successfully developed a simulation model in collaboration with managers and staff and evaluated it using real patient data. The evaluation covered resource planning for the summer vacation and among investigated scenarios, the most preferable scenario was easily identified without affecting daily work at the clinic.

Limited resources and high workload in a modern RT should not be a limiting factor for identifying new resource planning strategies around longer vacation periods or other changes in resource availability. There are, to our knowledge, no published guidelines on how to plan a vacation period for RT to avoid overtime/build-up of patients waiting to initiate treatment/maximize linac use. A PubMed search on June 9th, 2021, gave one relevant hit on various combinations of “capacity”, “vacation”, “planning”, “reduction” and “radiotherapy” with the relevant hit only describing a need in extra capacity to keep waiting times low while compensating missed fractions by treating twice a day due to bank holidays [11]. Our study contributes in this area by a novel simulation model over the RT process that enabled time-efficient evaluation of nine different scenarios with varying capacity settings and timing between workflow parts for the summer vacation period, without any impact on the daily clinical use. Notably, if strategies for the summer vacation period were to be evaluated only once per year in reality, it would take several years finding a promising strategy. Using simulations, this can be achieved within a few hours once a suitable model is available. Managers are expected to balance workload and patient waiting times and with a strategy using simulations, they can be aided in planning for the future [3], [7]. To overcome problems with many results from simulation models never being implemented in reality [12], we involved managers and booking staff in model development. Our approach included close collaboration with RT managers and staff both during development and when simulating the different scenarios. Active participation from the department has been reported to be a major factor for a successful implementation of new methods when facilitating change processes in healthcare using simulations [13].

With the assistance of the simulation model, we could identify strategies to reduce number of patients waiting to start treatment with completed preparations, to limit the need for overtime in the beginning of the treatment vacation period and to enable high linac utilization on both sides of the vacation period. To utilize linacs after the vacation period, the preparatory part needs adequate capacity close to the end of the treatment vacation period. However, having a small over-capacity in the preparations during the whole vacation period would not be a clinically-accepted strategy, since patients with completed preparations need to start within a short time limit. With delays between pre-treatment imaging and treatment start, changes in patient inner or outer anatomy can occur due to factors like tumor growth and weight loss. Even if not necessarily affecting treatment outcome [4], this can still cause psychological stress in patients [14]. The problems with overtime at the beginning of the treatment period, identified at some of the scenarios, can probably be limited with a well-thought-out patient booking strategy, where many patients are scheduled to end their treatment just before the vacation period starts. However, such a strategy also requires the matching of patients with a suitable medical priority ready for treatment, which in turn can generate an uneven workload at the preparation part if many patients with same number of fractions must start during a short period of time. According to our simulations, shorter vacation periods for the investigated department, four or six weeks compared to eight weeks, are not beneficial regarding workload and linac utilization and would require detailed planning with uneven capacity between the weeks to match all needs.

One strength in our strategy is the use of real data for model development which originate from the same department as the model is applied on. We also included the whole RT process within the same model to allow for an understanding of effects at both the preparation part and the treatment part as well as their interrelations. One limitation is that not all individual tasks in the preparatory part are evaluated separately, instead our approach combined mould, imaging, contouring, treatment planning and QA into one overall preparatory task given that excessive details about all steps would disguise the overall trends we aimed to identify. We neither included details on specific treatment techniques nor linac maintenance/downtime. Simulations were made with a fixed pre-treatment throughput time of 1.5 weeks, but changing the settings to 1 or 2 weeks had no impact on the overall results (data not shown). Since treatment techniques and other policies affecting the RT workflow was considered equal between the different simulated scenarios, addition of these kinds of variables are unlikely to have changed the overall message of this work but would have affected the actual output values to some degree. Detailed simulation of all individual steps could, however, be useful for understanding the interrelationships between the steps of the preparatory part. It needs to be kept in mind that simulation models developed to illustrate overall effects of a system or a process are not exact tools to plan associated production in detail. Specific quantitative results, e.g. number of patients waiting and capacity, are not for direct use, but the behavior between different evaluated scenarios and overall systemic trends are. For the purpose of strategic planning and to evaluate different scenarios, results using a simulation model can be helpful in a mid to long-term resource planning process. Our developed simulation model could, in its current version, be used by other RT departments since the simplified RT workflow used in the model is likely to be similar for any modern RT department and is available upon request. Different department sizes can be handled by using the adjustable capacity levels. However, input data are department specific since patient referral patterns vary as well as case mix of patients and different fractionation schedules which means that principles for pre-processing of data need to be applied on site before model use. For clinical implementation of any modelling results, including those identified in this study, careful evaluation of patient safety, staff satisfaction, and other quality aspects must be undertaken to assure both that implemented changes provide the intended positive effect but also that they do so without compromising RT from any other aspect. As a final remark, our model is prepared for the use of system dynamics, a methodology which offers additional possibilities in future research and clinical work to consider effects of dynamic complexity. Such possibilities include the addition of qualitative variables and feedback loops to the model as well as how changes in other treatment policies than those investigated here can affect resource planning. Qualitative variables could, for instance, quantify the abovementioned quality aspects and their effect on productivity. The feedback loops handle potentially nonlinear relationships between these variables. Our aim for further model development is, however, to provide functionality to solve additional problems raised by the department rather than to build an extensive model that may be unnecessarily complex for requested scenarios.

In conclusion, a simulation model over the RT process can aid managers with their resource planning decisions. We found that several scenarios for the planning of resource use around a longer vacation period easily could be evaluated and compared without affecting the clinical work. Starting the vacation period for the preparatory part one-two weeks prior to the treatment part was identified as most beneficial for the investigated department and use case, a strategy which could be useful for other RT departments too. With a simulation model of the whole RT process at hand, managers get a systemic understanding about effects of various changes and can take well-informed strategic decisions about how to be prepared for future events at their department.

## Data availability statement

Research data are stored in an institutional repository and are available from the corresponding author, upon reasonable request.

## Declaration of Competing Interest

The authors declare that they have no known competing financial interests or personal relationships that could have appeared to influence the work reported in this paper.
