# Supplementary material for: Resource planning principles for the radiotherapy process using simulations applied to a longer vacation period use case
Source: Tech Innov Patient Support Radiat Oncol. 2021 Oct 16;20:17–22. doi: 10.1016/j.tipsro.2021.10.001 (PMC8524937; doi:10.1016/j.tipsro.2021.10.001)
Supplement: Supplementary Data 1 [file mmc1.pdf]

Description of the simulation model

This is a continuous simulation model covering the whole radiotherapy process. Inflow data and information on number of fractions per workflow group are imported through linked Excel-sheets. The overall model structure is shown in Figure A1 and is directed from left to right. The simulation model starts with patient referrals (*Inflow*) and then patients enter the waiting list to start preparations (*Patients waiting preparation*). When preparation capacity is available, the patient moves into the preparation phase (*Preparations*) and remains there for 1.5 week (similar time as current clinical booking templates). When the preparation part is completed, patients move forward to the waiting list for starting treatment (*Wait start treatment*). When treatment capacity is available, the patients enter treatment (*Under treatment*) and remains until all fractions are delivered. When the treatment is completed, the patient exits the model (*Treatment completed*).

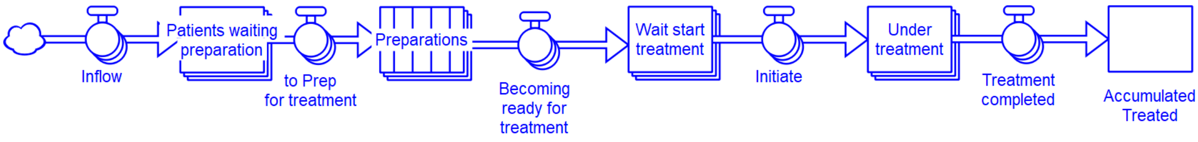

Figure A1. Overview of the simulation model.

The treatment part is more detailed and consists of four equal sections with 10 fractions in each section (maximum number of fractions in the current version of the model is 40), the first section for fractions 1-10 is shown in Figure A2. When the treatment is initiated, patients start to wait for their first fraction (*Wait frac 1*) and when this is completed, they move forward in the model with one fraction given each simulated day (five treatments per week). Patients continue in the model until they reach the prescribed number of fractions after which they exit the treatment part. Treatment capacity is limited and controlled from the user interface. Capacity need is calculated to allow all patients with ongoing treatments to have one fraction per day. If there is available capacity after the ongoing patients have been considered, new patients are allowed to start treatment.

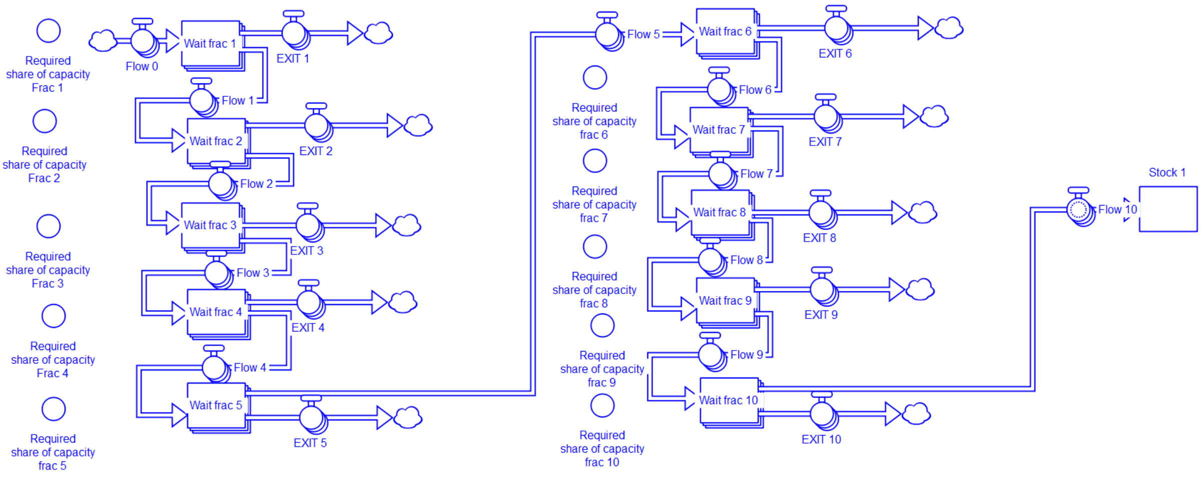

Figure A2. Details on the first 10 fractions. The treatment step includes four main variables/steps: *Required share of capacity Frac n* is a calculation of the capacity need for all patients for each fraction *n*, to be summed for a complete need in resources for all fractions and allow new patients to start if there is capacity left. *Wait frac n*, patients in waiting for their next fraction (*n+1*). *Flow n*, the

treatment where patients receive radiation. *EXIT n*, patient exits after fraction *n* if *n* equals the total number of planned fractions. The index *n* stands for any fraction between first and last according to the patient's planned number of fractions.

The user interface used for evaluating different scenarios allows adjustment of variables and is shown in Figure A3.

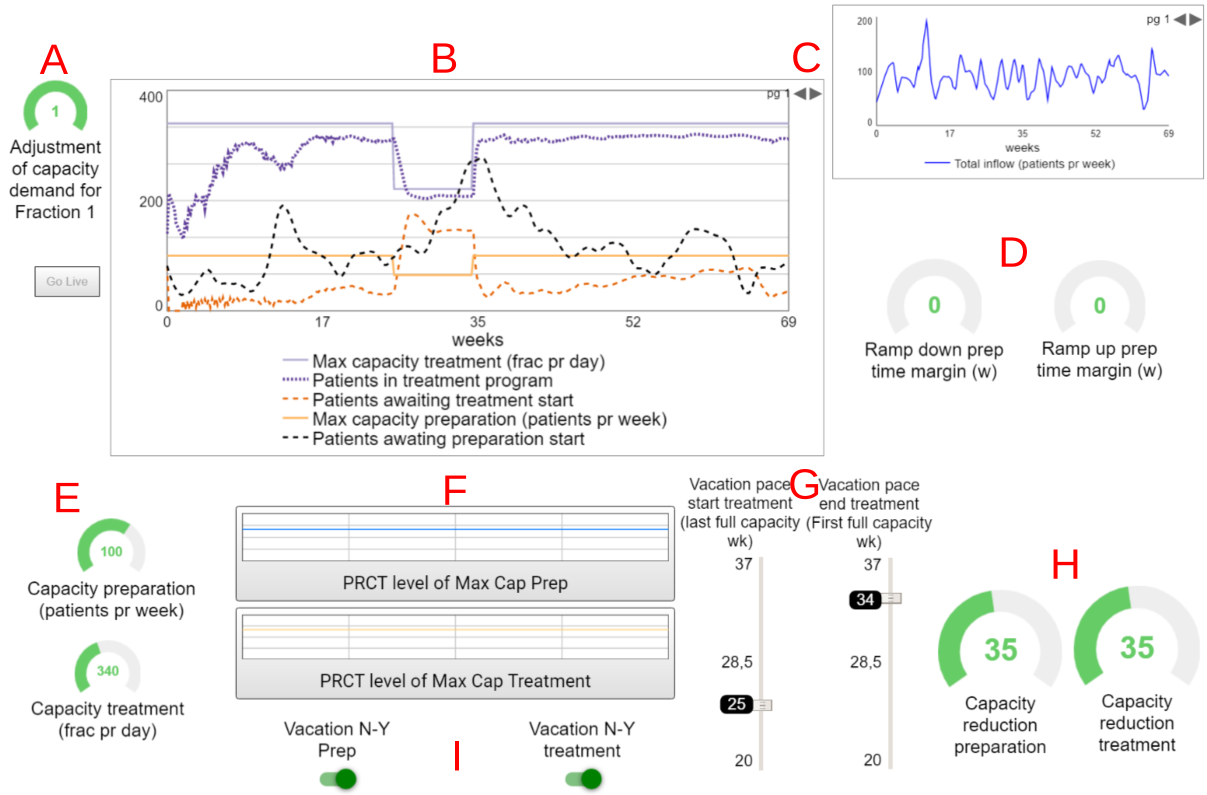

Figure A3. View of the front-end interface used for simulations. Simulation model settings are adjustable from this view.

A: Capacity demand for the first fraction, where 0.5 mean additional demand of 50% compared to successive fractions and 1 means 100% extra (doubled).

B: Simulation results, showing maximum allowed capacity for the two parts (purple: linac, yellow: preparation), number of patients under treatment (purple), patients waiting to start treatment with completed preparations (orange) and number of patients waiting to start preparations (black).

C: Total referral (inflow) pattern (patients per week).

D: Adjustment of start and end weeks of the preparation part in relation to the treatment part.

E: Baseline capacity for number of preparations possible each week and capacity for number of treatment fractions per day.

F: Manual adjustment of capacity reduction.

G: Vacation period start and end week.

H: Capacity reduction for preparation and treatment, respectively.

I: Enable/disable capacity reduction.
